# TME5/354: A Videoconference System for Telepathology

**DOI:** 10.2196/jmir.1.suppl1.e112

**Published:** 1999-09-19

**Authors:** M de Cabo, I Dimitriadis, E Fernández, N Pérez

**Affiliations:** ^1^Valladolid UniversityValladolidSpain

**Keywords:** Telemedicine, Telepathology, Videoconference

## Abstract

**Introduction:**

In this abstract we introduce an application of computer based collaborative work (CSCW) developed in videoconference to offer telepathology services. The application is focused on collaboration between a GP (General Practitioner) from a primary health centre and the specialist of pathological anatomy section from the HMC (Hospital de Medina del Campo), with the purpose of sharing data, tissue images and opinions. This work forms part of a bigger project financed by CICYT, the Government Commission of Science and Technology of Spain with TEL97-0750. The aim of this part of the project was to develop a new application of teleconferencing based on the NetMeeting SDK (Software Development Kit). Because current teleconferencing systems have been developed for common purposes they don't fulfil the requirements found out for pathologists. A new user interface was designed to overcome this problem.

**Methods:**

After many queries to the physicians and managers of the HMC, we found out the functions that our interface should offer:

- Seeing and talking to other physicians and/or patients.
- Sharing a digital image previously recorded in the physician's PC.
- Sharing patient records.
- Sharing pre-recorded videos from a digital microscope.
- Sending files to another physician for analysing in a later instance, thus minimising delays on a real time transmission.
- Sending e-mail about issues to be discussed in the videoconference session.
- Working with moving images from the microscope to establish which area it is the most interesting one.
- Having a written chat for recording it and for saving bandwidth (voice uses more bandwidth).

The system will be implemented in a particular environment that raises some issues on particular requirements about the topology, elements and media of transmission over the network, the operating system to be used, the quality of service, the functionality and the cost.

**Results:**

The application was proved in two different scenarios (LAN and telephone lines) to compare delays caused by the transmission systems. The present system has been initially exposed to internal users (pathologist of HMC) with positive response. A systematic evaluation will be performed together with other parts of the project.

**Discussion:**

The major benefit of the introduced interface is that it facilitates for the pathologist the use of videoconference as this is adapted to his own needs. Another benefit is that rural areas are nearer (not physically but functionally) to the services of pathological services.There is still a need for further improvement of the system. This improvement can be reached with the following alternatives:

- Better computer resources, regarding processing capability and image capturing.
- Better communication resources
- Developing of the videoconference system from scratch, instead of using NetMeeting and its limitations to pathology requirements

